# Bioactive peptide design using the Resonant Recognition Model

**DOI:** 10.1186/1753-4631-1-7

**Published:** 2007-07-19

**Authors:** Irena Cosic, Elena Pirogova

**Affiliations:** 1 School of Electrical and Computer Engineering, RMIT University, GPO Box 2476V Melbourne, Victoria, 3001, Australia

## Abstract

With a large number of DNA and protein sequences already known, the crucial question is to find out how the biological function of these macromolecules is "written" in the sequence of nucleotides or amino acids. Biological processes in any living organism are based on selective interactions between particular bio-molecules, mostly proteins. The rules governing the coding of a protein's biological function, i.e. its ability to selectively interact with other molecules, are still not elucidated. In addition, with the rapid accumulation of databases of protein primary structures, there is an urgent need for theoretical approaches that are capable of analysing protein structure-function relationships. The Resonant Recognition Model (RRM) [1,2] is one attempt to identify the selectivity of protein interactions within the amino acid sequence. The RRM [1,2] is a physico-mathematical approach that interprets protein sequence linear information using digital signal processing methods. In the RRM the protein primary structure is represented as a numerical series by assigning to each amino acid in the sequence a physical parameter value relevant to the protein's biological activity. The RRM concept is based on the finding that there is a significant correlation between spectra of the numerical presentation of amino acids and their biological activity. Once the characteristic frequency for a particular protein function/interaction is identified, it is possible then to utilize the RRM approach to predict the amino acids in the protein sequence, which predominantly contribute to this frequency and thus, to the observed function, as well as to design *de novo *peptides having the desired periodicities. As was shown in our previous studies of fibroblast growth factor (FGF) peptidic antagonists [2,3] and human immunodeficiency virus (HIV) envelope agonists [2,4], such *de novo *designed peptides express desired biological function. This study utilises the RRM computational approach to the analysis of oncogene and proto-oncogene proteins. The results obtained have shown that the RRM is capable of identifying the differences between the oncogenic and proto-oncogenic proteins with the possibility of identifying the "cancer-causing" features within their protein primary structure. In addition, the rational design of bioactive peptide analogues displaying oncogenic or proto-oncogenic-like activity is presented here.

## Background

Bioengineering has emerged as a truly multidisciplinary field with the aim of improving the quality of human health through increased biological and medical knowledge facilitating the development of novel devices and drugs. The ability to predict the functions and three-dimensional shapes of biological molecules would certainly be useful in designing such therapeutic drugs. Some approaches attempt to interrupt the processes occurring in biochemical pathways in the diseased state by causing a key molecule to stop functioning. Drugs may be designed that bind to the active region and inhibit this key molecule. Most of the drugs that exist today were discovered by chance, or by trial and error. Unlike this historical method of drug discovery, by testing of chemical substances on animals and matching the apparent effects to treatments, a rational drug design begins with knowledge of specific chemical responses in the body or target organism, and tailoring combinations of these to fit a treatment profile. Though there have been many attempts at such designs in recent years, they have encountered major hurdles, and it is only now that the techniques involved seem ready to produce practical results.

However, such drugs would also have to be designed in such a way as not to affect other important molecules that may be similar in appearance to the key molecules. Sequence homologies are often used to identify such risks. The structure of the drug molecule that can specifically interact with the biomolecules can be modeled using computational tools. These tools can allow a drug molecule to be constructed using knowledge of its structure and the nature of its active site. However, many of these approaches are hindered by the practical problems of chemical synthesis. It has been also suggested to use large and proteinaceous in nature molecules as drug molecules instead of small chemical molecules. There have also been suggestions to make these complex molecules using messenger ribonucleic acid (mRNA) and to employ gene silencing in therapeutic applications.

Currently, significant scientific effort is being directed to solve the problem of finding a cure for cancer. New and advanced drugs and methodologies have been developed and applied with some degree of success, however, the battle with cancer still continues. There is an urgent need for theoretical approaches that are capable of analysing protein and DNA structure-function relationships leading to the design of new drugs capable of fighting diseases such as cancer. The RRM [1,2] represents a non-traditional computational approach designed for structure-function analysis of protein and deoxyribonucleic acid (DNA) sequences and their mutual interactions.

Proteins are polymers built up from amino acids. The great diversity and versatility of protein sequences are derived from the properties of the twenty different amino acid side chains that may exist in a protein molecule and which are reflected in the wide range of bioactivity of the formed protein molecules. However, proteins are able to express their biological functions only by achievement of a certain active native conformation, the so-called three-dimensional structure (3-D). Obviously, the particular function of a given protein and its active 3-D structure are determined by the sequence of amino acids forming this particular protein molecule. The protein's biological function is encrypted within the protein's primary structure, i.e. the sequence of amino acids. There have been many attempts to discover the main principles governing the functional behaviour of proteins. Typical approaches are either homology characterisation of specific features of the primary and secondary structure of proteins, or molecular modelling of the protein's 3-D structure. Although such approaches permit a significant insight into the protein's structure and active site location, they still do not provide sufficient knowledge about informational, structural and physicochemical parameters crucial to the selectivity of protein interactions which can be used for the *de novo *design of peptide analogous with desired biological activity [1,2].

The RRM model [1,2], employed in this study, essentially belongs to the approaches able to gain the protein's functional and structural information by the analysis of the amino acid sequences and DNA. The RRM is a physical and mathematical model that interprets the protein's sequence linear information using the signal analysis method of Fourier and Wavelet Transforms. It has been found through extensive research that proteins with the same biological function have a common frequency component in their numerical spectra. This frequency was found then to be a characteristic feature for particular protein biological function or interaction [1,2]. Once this characteristic frequency is identified, it is possible then to utilize the RRM to predict the amino acids in the sequence that predominantly contribute to this frequency and consequently to the observed function. Also it becomes possible to design peptides having the desired periodicities.

It has been proposed [1,2] that RRM frequency represents a unique parameter of the interacting sequences and thus, characterises their mutual interaction. In our previous work it has been also proposed that this characteristic frequency could represent the oscillations of a physical field, which are responsible for information transfer between the interacting bio-molecules [1,2]. Consequently, it is postulated that the RRM characteristic frequency is a relevant parameter for mutual recognition between bio-molecules and is significant in describing the interaction between proteins and their substrates or targets. Therefore, it can be concluded that the RRM characteristic frequency may dictate the selectivity of protein interactions.

In this paper we discuss in more detail the mathematical and physical background of the RRM model and its successful application to bioactive peptide design and protein active site predictions. In order to design biologically active peptides it is of primary importance to determine which amino acids are responsible for the biological activity of native proteins. Essentially, systematic mutation studies would result in the generation of enormous numbers of peptides which should be subsequently synthesised and tested to find those peptides that are biologically active. Here we present the results of our computational analysis of oncogene and proto-oncogene proteins and the rational design of bioactive peptide analogues having the oncogenic or proto-oncogenic-like activity.

## Methods

### Resonant Recognition Model

Since the binding of therapeutic peptides to appropriate receptors in the body occurs through intermolecular interactions, the ability to determine which amino acids are responsible for biological activity is essential. With the rapid expansion of the protein databases, the identification of the biological function of newly sequenced proteins or the determination of their relationships with defined functional families becomes a real problem. Therefore, the introduction of additional information concerning the relationship between amino acids within the protein sequence would be helpful. The information encoded in the amino acid sequence ultimately determines the 3-D structure and biological function of a protein under physiological conditions. In order to understand empirical relationships between the amino acid sequence, structural patterns and functional sites, the RRM has been invented. The application of the RRM involves two stages of calculation. The first is the transformation of the amino acid sequence into a numerical sequence. Each amino acid is represented by the value of the Electron-Ion Interaction Potential (EIIP) describing the average energy states of all valence electrons in a given amino acid. The EIIP values for each amino acid were calculated using the following general model of pseudo-potentials [8]:

〈k+q|w|k〉=0.25Zsin⁡(1.04∗πZ)2π
 MathType@MTEF@5@5@+=feaafiart1ev1aaatCvAUfKttLearuWrP9MDH5MBPbIqV92AaeXatLxBI9gBaebbnrfifHhDYfgasaacH8akY=wiFfYdH8Gipec8Eeeu0xXdbba9frFj0=OqFfea0dXdd9vqai=hGuQ8kuc9pgc9s8qqaq=dirpe0xb9q8qiLsFr0=vr0=vr0dc8meaabaqaciaacaGaaeqabaqabeGadaaakeaadaaadaqaaiabdUgaRjabgUcaRiabdghaXnaaemaabaGaem4DaChacaGLhWUaayjcSdGaem4AaSgacaGLPmIaayPkJaGaeyypa0JaeGimaaJaeiOla4IaeGOmaiJaeGynauZaaSaaaeaacqWGAbGwcyGGZbWCcqGGPbqAcqGGUbGBcqGGOaakcqaIXaqmcqGGUaGlcqaIWaamcqaI0aancqGHxiIkiiGacqWFapaCcqWGAbGwcqGGPaqkaeaacqaIYaGmcqWFapaCaaaaaa@4E61@

where *q *is the change of momentum of the delocalised electron in the interaction with potential w, while:

Z=∑iZN
 MathType@MTEF@5@5@+=feaafiart1ev1aaatCvAUfKttLearuWrP9MDH5MBPbIqV92AaeXatLxBI9gBaebbnrfifHhDYfgasaacH8akY=wiFfYdH8Gipec8Eeeu0xXdbba9frFj0=OqFfea0dXdd9vqai=hGuQ8kuc9pgc9s8qqaq=dirpe0xb9q8qiLsFr0=vr0=vr0dc8meaabaqaciaacaGaaeqabaqabeGadaaakeaacqWGAbGwcqGH9aqpdaWcaaqaamaaqafabaGaemOwaOfaleaacqWGPbqAaeqaniabggHiLdaakeaacqWGobGtaaaaaa@34E9@

where Z is the number of valence electrons of the i-th component of each amino acid and N is the total number of atoms in the amino acid. A unique number can thus represent each amino acid or nucleotide, irrespective of its position in a sequence. Numerical series obtained in this way are then analysed by digital signal analysis methods in order to extract information relevant to the biological function. As the average distance between amino acid residues in a polypeptide chain is about 3.8 Å, it can be assumed that the points in the numerical sequence derived are equidistant. For further numerical analysis the distance between points in these numerical sequences is set at an arbitrary value d = 1. Then the maximum frequency in the spectrum is *f*_max _= 1/2d = 0.5. The total number of points in the sequence influences the resolution of the spectrum only. Thus, for an N-point sequence the resolution in the spectrum is equal to 1/N. The n-th point in the spectral function corresponds to the frequency f = n/N. In order to extract common spectral characteristics of sequences having the same or similar biological function, the following cross-spectral function was used:

S_n _= X_n_Y_n_*   n = 1,2,..., N/2

where X_n _are the Discrete Fourier Transform (DFT) coefficients of the series x(n) and Y_n_* are complex conjugate discrete Fourier transform coefficients of the series y(n). Peak frequencies in the amplitude cross-spectral function define common frequency components of the two sequences analysed. To determine the common frequency components for a group of protein sequences, the absolute values of multiple cross-spectral function coefficients M have been calculated as follows:

|M_n_| = |X1_n_|.|X2_n_|...|XM_n_|   n = 1,2,..., N/2

Peak frequencies in such a multiple cross-spectral function denote common frequency components for all sequences analysed. Signal-to-noise ratio (S/N) for each peak is defined as a measure of similarity between sequences analysed. S/N is calculated as the ratio between signal intensity at the particular peak frequency and the mean value over the whole spectrum. The extensive experience gained from previous research [1,2,8-10] suggests that a S/N ratio of at least 20 can be considered as significant. The multiple cross-spectral functions for a large group of sequences with the same biological function have been named the "consensus spectrum". The presence of a peak frequency with significant signal-to-noise ratio in a consensus spectrum implies that all of the analysed sequences within the group have one frequency component in common. This frequency is related to the biological function provided the following criteria are met:

• Only one peak exists for a group of protein sequences sharing the same biological function.

• No significant peak exists for biologically unrelated protein sequences.

• Peak frequencies are different for different biological functions.

In our previous studies, the above criteria have been tested with over 1000 proteins from 25 functional groups [1,2]. The following fundamental conclusion was drawn from our studies: one RRM peak frequency characterises one particular biological function or interaction [1,2]. Therefore, those peaks are named as the RRM characteristic frequencies. It was shown that proteins and their interacting targets (receptors, binding proteins, and inhibitors) display the same characteristic frequency in their interactions. However, it is obvious that one protein can participate in more than one biological process, i.e. revealing more than one biological function. Despite proteins and their targets have different biological functions they can participate in the same biological process. Therefore, it has been postulated that interacting molecules "communicate" with each other, i.e. recognise each other at a distance, on the basis of the same/similar (within the calculation error) characteristic frequency but opposite phases at that frequency [2].

However, to appreciate the significance of the RRM characteristic frequency it is important to explore what is meant by the biological function of proteins. As pointed out above, each biological process is driven by proteins that selectively interact with other proteins, DNA regulatory segment or small molecules. These interactive processes involve energy transfer between the interacting molecules that are highly selective. The question arises how is this selectivity achieved? In the RRM it is postulated that the selectivity is defined within the amino acid sequence. It has been shown that proteins and their targets share the same characteristic frequency but are opposite in phase [1,2] for each pair of interacting macromolecules. Thus, we conceptualise that RRM characteristic frequencies represent proteins general functions as well as mutual recognition between a particular protein and its target (receptor, ligand, etc). As this recognition arises from the matching of periodicities within the distribution of energies of free electrons along the interacting proteins, it can be regarded as the resonant recognition. The RRM model assumes that characteristic frequencies are responsible for the resonant recognition between macromolecules at a distance. Thus, these frequencies have to represent oscillations of some physical field which can propagate through water dipoles. One possibility is that this field is electromagnetic in nature [1,2].

There is evidence that proteins and DNA have certain conducting properties [12]. If so, then charges would be moving through the backbone of the macromolecule and passing through different energy stages caused by the different side groups of various amino acids or nucleotides. This process provides sufficient conditions for the emission of electromagnetic waves. Their frequency range depends on the charge velocity, which in turn depends on the nature of charge movement (superconductive, conductive, soliton transfer, etc.) and on the energy of the field that causes charge transfer. The nature of this physical process is still unknown; however, some models of charge transfer through the backbone of macromolecules have been accepted [13-15]. To simplify the calculations, we assumed the electron transfer is attributed to the difference of the free EIIP at the amino group (NH_2_) and carboxyl (COOH) group of the protein molecule. According to the theory of pseudo-potentials [16], this potential energy difference is:

*W = W(COON) - W(NH_2_) = 0.13 Ry*

This energy difference allows for a maximum velocity of the electrons of:

Vmax⁡=2eW/m
 MathType@MTEF@5@5@+=feaafiart1ev1aaatCvAUfKttLearuWrP9MDH5MBPbIqV92AaeXatLxBI9gBaebbnrfifHhDYfgasaacH8akY=wiFfYdH8Gipec8Eeeu0xXdbba9frFj0=OqFfea0dXdd9vqai=hGuQ8kuc9pgc9s8qqaq=dirpe0xb9q8qiLsFr0=vr0=vr0dc8meaabaqaciaacaGaaeqabaqabeGadaaakeaacqWGwbGvdaWgaaWcbaGagiyBa0MaeiyyaeMaeiiEaGhabeaakiabg2da9maakaaabaWaaSGbaeaacqaIYaGmcqWGLbqzcqWGxbWvaeaacqWGTbqBaaaaleqaaaaa@3853@

where e is the electron charge, and m is electron mass. Therefore

*V *< 7.87·10^5 ^m/sec

As an inherent assumption is the amino acids in the protein sequence are equidistant at:

*d = 3.8 Å*

Then, the maximum frequency that could be emitted during the electron transfer is

*F*_max _<*V*/2*d*

or

*F*_max _< 10^15 ^Hz

while the corresponding wavelength is

*L*_min _> 330 nm

The minimum frequency that could be emitted depends on the total length of the protein

*F*_min _= 2 *F*_max_/*N*

where *N *is the total number of amino acids in the protein. For example with proteins of 200 amino acids in length, the minimum frequency is

*F*_min _< 10^13 ^Hz

and the corresponding wavelength is

*L*_max _< 30 000 nm

This range from 30000 nm to 300 nm is very wide, starting from the very low infrared through the visible to the ultraviolet regions [1,2].

To establish the possibility that macromolecular interactions are based on the resonant energy transfer between interacting molecules, we compared our computational predictions with a number of published experimental results (Table 1). This assumption has been studied for a number of examples that include:

**Table 1:** The RRM frequencies and characteristic absorption frequencies of different visible light-absorbing protein groups and their scaling factor, K [2]

protein	nm	rrm	cm-1	K
cyt c	415	0.473	24096.3855	196
blue	430	0.475	23255.814	204
green	540	0.355	18518.5185	191
red	570	0.346	17543.8596	197
hem.	14770	0.02	677.04807	295
purple	860	0.281	11627.907	241
flavodoxin	470	0.379	21276.5957	178
igf	400	0.492	25000	196
fgf	441.6	0.453	22644.9275	200
insulin	552	0.344	18115.942	189
growth f.	633	0.293	15797.7883	185
	650	0.293	15384.6154	190
pdgf	830	0.242	12048.1928	200
chymotr.	851	0.236	11750.8813	200
calculative	400	0.5	25000	200

• Laser light growth promotion of cells using the particular frequencies of light producing a similar effect to that due to the presence of growth factor proteins [17],

• Chymotrypsin activation (increase of enzyme activity) achieved by laser light radiation in the range 850–860 nm [18],

• Activation of highly homologous plant photoreceptors which, although being very homologous, absorb different wavelengths of light [19],

• Photo-activated proteins (e.g. rhodopsins, flavodoxins etc.), that absorb light through the prosthetic group but where frequency selectivity of this absorption and consequent activation is defined by the protein sequence [20,21].

**Figure 1:**
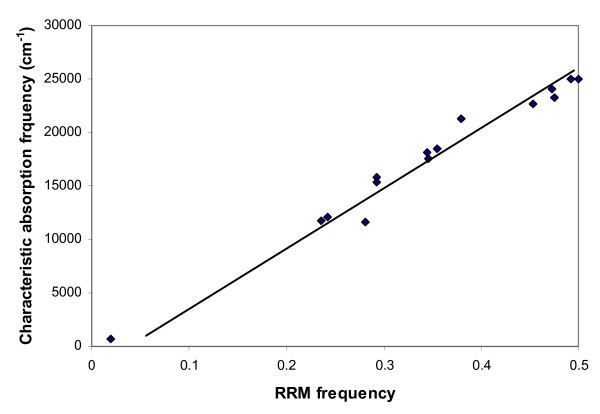
Linear correlation between RRM frequencies and corresponding absorption frequencies of different visible light-absorbing protein groups.

All these results imply that specificity of protein interactions is based on the resonant electromagnetic energy transfer at the specific frequency, observed for each interaction. The numerical frequencies that have been obtained similarly by the RRM for various other groups of visible light-absorbing proteins are compared with their corresponding characteristic absorption frequencies in Table 1 and this linear correlation is shown in Figure 1[1,2]. A result of considerable significance is that the scaling factor between these two sets of data is almost constant about the value of K = 200 [1,2]. Thus, a strong linear correlation appears to exist between the numerical characteristic frequencies defined by the RRM and the experimentally determined frequencies corresponding to the electromagnetic radiation absorption of such proteins. From this correlation it can be observed that the full range of wavelengths, which can be related to RRM characteristic frequencies, is over 400 nm. This finding is in complete accord with the frequency range previously associated to the RRM spectra and calculated from the charge velocities through the protein backbone. It can be now inferred from both correlations that approximate wavelengths in real frequency space can be calculated from the RRM characteristic frequencies for each biologically related group of sequences. Furthermore, these calculations can be used to predict the wavelength of visible and near-infrared irradiation which may produce a biological effect [14,15].

Once the characteristic frequency for the particular biological function or interaction is determined, it becomes possible to identify the individual "hot spot" amino acids that contributed most to this specific characteristic frequency and thus, possibly to the observed biological behaviour of the protein.

### "Hot spots" in terms of the RRM and 3-D protein structures

It is known that proteins cannot express their biological function until they achieve a certain active 3-D conformation. By identifying the characteristic frequency of a particular protein, it is possible to predict then which amino acids in the sequence predominantly contribute to the frequency and consequently to the observed function [1,2,8,9]. Since the characteristic frequency correlates with the biological function, the positions of the amino acids that are most affected by the change of amplitude at the particular frequency can be defined as "hot spots" for the corresponding biological function. The strategy for this prediction includes the following steps:

• To determine the unique characteristic frequency for the specific biological function by multiple cross-spectral analysis for the group of sequences with the corresponding biological function.

• To alter the amplitude at this characteristic frequency in the particular numerical spectrum. The criterion used for identifying the critical characteristic frequency change is the minimum number of "hot spot" amino acids that are least sensitive to further changes in the amplitude of the characteristic frequency.

• To derive a numerical sequence from the modified spectrum using DFT.

**Figure 2:**
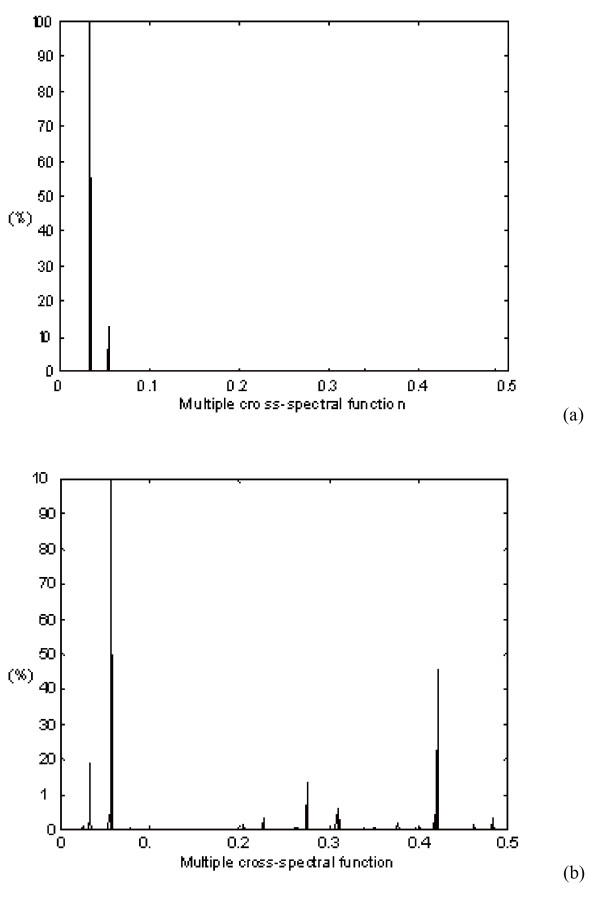
Multiple cross-spectral function of: a) oncogene and b) proto-oncogene proteins. The prominent peak(s) denote common frequency components. The abscissa represents RRM frequencies, and the ordinate is the normalized intensity.

It is known that a change in amplitude at one frequency in the spectrum causes changes at each point in the numerical sequence. Thus a new numerical series is obtained where each point is different from those in the original series. Detecting the amino acids corresponding to each element of this new numerical sequence can then be achieved using tabulated values of the EIIP or other appropriate amino acid parameters. The amino acids in the new sequence that differs from the original ones reside at the points mostly contributing to the frequency. These hot spots are related to this frequency and to the corresponding biological function. The procedure described was used in a number of examples: IL-2 [9], hemoglobins, myoglobins and lysozymes [8], chymotrypsins [12], glucagons, TNFs [10], EGFs, FGFs [2]. These examples have shown that such predicted amino acids denote residues crucial for protein functions. Consequently, these "hot spot" amino acids are found spatially clustered in the protein's 3-D structure in and around the protein active site. As these specific amino acids most strongly influence the characteristic frequency, their cluster does represent a site in the protein where the signal of the characteristic frequency for the specific protein property is dominant. As this cluster of amino acids has been found positioned in and around the active site (Fig. 2), it is proposed that these specific amino acids play a crucial role in determining the structure of the active site and possibly, the active structure of the whole molecule [8,11].

### Bioactive Peptide Design

Following the determination of the RRM characteristic frequencies and corresponding phases for particular biological functions, it is possible to design amino acid sequences having those spectral characteristics only. It is expected that the designed peptide will exhibit the desired biological activity. The strategy for the design of such defined peptides is as follows:

1) In the multiple cross-spectral function of the group of protein sequences sharing the corresponding biological function to determine the unique RRM frequency that characterises this specific biological function/interaction.

2) To define the characteristic phases at the characteristic frequencies for the particular protein that is chosen as the parent for agonist/antagonist peptide design.

3) To derive a numerical sequence from the known characteristic frequencies and phases. This can be done by summing sinusoids of the particular frequencies, amplitudes and phases. The length of the numerical sequence is defined by the appropriate frequency resolution, and the required peptide's length.

4) To determine the amino acids that corresponds to each element of the new numerical sequence. It can be achieved by the tabulated EIIP or other appropriate amino acid parameters [1,2,10].

In our previous research these procedures have been applied to different examples of proteins [3,4,7], aiming at the design of peptide analogous having the same functional activity as their parent proteins:

• The RRM has been applied to structure-function studies with basic fibroblast growth factor (bFGF) [3]. Property-pattern characteristics for biological activity and receptor recognition for a group of FGF-related proteins were defined and then used to aid the design of a set of peptides which can act as bFGF antagonists. Molecular modelling techniques were then employed to identify the peptide within this set with the greatest conformational similarity to the putative receptor domain of bFGF. The 16 mer peptide, which exhibits no sequence homology to bFGF, antagonised the stimulatory effect of bFGF on fibroblast [^3^H]thymidine incorporation and cell proliferation, but exerted no effect itself in these in vitro bioassays [3].

• The interaction between HIV virus envelope proteins and CD4 cell surface antigen has a central role in the process of virus entry into the host cell. Thus, blocking the interaction between the envelope glycoproteins and CD4 surface antigen, known to be the HIV receptor, should inhibit infection [4]. For this purpose, six peptides, each 20 amino acids in length, were designed using the RRM procedure. To validate the RRM predictions, the peptides designed were evaluated experimentally [4]. These investigations were performed initially by evaluating the reactivity and cross-reactivity of all such designed peptides with the corresponding antibodies [4]. The results obtained have shown significant cross-reactivity to the polyclonal antibodies raised against peptides that share at least one characteristic frequency and phase at this frequency. The results provide experimental confirmation of the concept that RRM frequency characteristics reflect important parameters associated with bio-molecular recognition and in particular antibody-antigen recognition.

In our previous study [7] the RRM was employed for the analysis of forty six oncogene and fifteen proto-oncogene proteins in determining the structure-function relationship among these sequences. The common frequencies have been determined computationally that represent the characteristic features of their common biological activity, i.e. the ability to promoter uncontrolled cell proliferation, in case of the oncogene proteins, and normal cell growth for proto-oncogenes. This study emphasises the *de novo *design of peptide analogues only on the basis of the frequencies and phases predicted computationally. Ultimately, these designed peptides would exhibit the desired oncogene or proto-oncogenic-like activity as their parental protein.

## Results and discussion

Cancer cells development is attributed to different mutations and alterations in DNA. DNA controls all cell biological activities. Each gene serves as a recipe on how to build a protein molecule. Proteins perform important tasks for the cell functions or serve as building blocks. The flow of information from the genes determines the protein composition and thereby the functions of the cell. Investigations of cellular and molecular mechanisms of uncontrolled cell proliferation associated with their transformation have resulted in the identification of a large number of genes (oncogenes), the products of which are involved in cell neoplasia.

The oncogenic family of proteins is generally involved in regulation of cell proliferation through regulation of DNA transcription. This regulation can be achieved indirectly via different signal pathways as it is in the case of tyrosine kinase oncogene products eg. *src, ros, fps, fes *and *yes *or directly by protein-DNA binding, as it is the case of nuclear proteins like *myc, fos *and *myb*. There is also a group of oncogene products including *ets-, sfi- and ras *– related proteins, for which *in vivo *biological functions and pathways of action have still to be fully defined [2,7,22,23]. The Ha-*ras *oncogene family (p21 proteins) are known to function *in vitro *as GTP binding proteins involved in signal transduction pathways, as well as in control of DNA synthesis, cell transformation, proliferation and differentiation [2,7,22,23]. Although it is known that the cellular, proto-oncogenic form of p21 is involved in control of cell proliferation, while the oncogenic form is additionally involved in cell transformation and differentiation, the exact mode of action and signal pathways of the Ha-ras p21 proteins (cellular, proto-oncogenic and oncogenic) still remain unknown [22,23].

The RRM approach has been applied here to a group of forty six oncogene proteins, with the aim of ascertaining their RRM frequency characteristics. The obtained RRM characteristic frequencies are as follows: f_1 _= 0.0322 ± 0.004, S/N = 297.29 and f_2 _= 0.0537 ± 0.004, S/N = 77.25. As is evident from Figure 2, both identified frequencies with significantly different amplitude ratios are observed in the cross-spectral function of all oncogene proteins. The prominent frequency component at f_1 _= 0.0322 ± 0.004, S/N = 297.29, common to the analysed sequences, characterises a common biological behaviour of this group of oncogene products, i.e. the ability to promote an uncontrolled cell growth and proliferation. The same RRM procedures have been applied for analysis of fifteen proto-oncogene sequences (Fig. 2). As can be observed from Figure 2 there is a prominent peak identified at f_2 _= 0.0537 ± 0.004, S/N = 199.74. This characteristic frequency determined represents a characteristic feature of all proto-oncogene sequences analysed and corresponds to their common biological behaviour, i.e. normal cell growth. It is noteworthy that the same frequency f_2 _= 0.0537 ± 0.004, S/N = 77.25 was identified as the less significant peak existing in multiple cross-spectral function of oncogene proteins. According to the RRM each specific biological function of the protein is characterised by a single frequency [7]. Thus, two peak frequencies identified within the RRM for forty six oncogene proteins f_1 _= 0.0322 ± 0.004, S/N = 297.29 and f_2 _= 0.0537 ± 0.004, S/N = 77.25, correspond to two different protein functions of these protein sequences.

In this study Ha-*ras *p21 sequence (Harvey Murine sarcoma virus) was used as a protein model for the peptide design. The RRM procedures have been applied to Ha-*ras *p21 protein to design the peptide that exhibits *ras*-like activity, i.e. ability to transform cells. The design of six *de novo *peptides A-F was based on two characteristic frequencies and phases determined for the entire functional group of oncogene proteins f_1 _= 0.0322, φ_1 _= 1.641 and f_2 _= 0.0537, φ_2 _= 2.460

**Figure 3:**
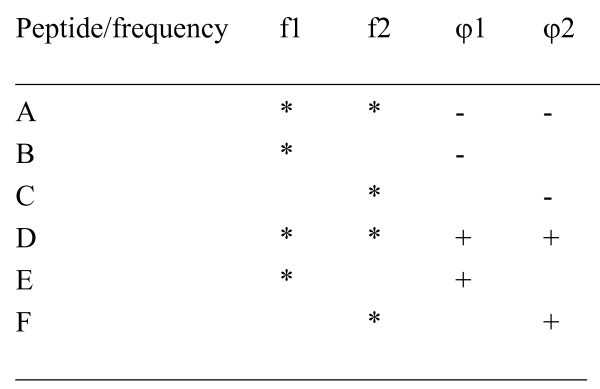
Schematic presentations of the designed peptides [4].

Each peptide has either one or both frequencies with the same or opposite phases at these frequencies as presented in the Fig 3.

Here the RRM has been applied to **Ha-*ras *p21 **protein sequence to design the peptide that will exhibit *ras*-like activity, i.e. ability to transform cells.

The original protein is presented below:

Transforming protein (H-ras) – Harvey murine sarcoma virus, 241 amino acids sequence

MPAARAAPAADEPMRDPVAPVRAPALPRPAPGAVAPASGGARAPGL

AAPVEAMTEYKLVVVGARGVGKSALTIQLIQNHFVDEYDPTIEDSYR

KQVVIDGETCLLDILDTTGQEEYSAMRDQYMRTGEGFLCVFAINNTK

SFEDIHQYREQIKRVKDSDDVPMVLVGNKCDLAGRTVESRQAQDLAR

SYGIPYIETSAKTRQGVEDAFYTLVREIRQHKLRKLNPPDESGPGCMSC

KCVLS

The peptide analogous has been designed on the basis of defined frequencies and phases:

f_1 _= 0.0322 φ_1 _= 1.641 (characteristic frequency of oncogene proteins)

f_2 _= 0.0537 φ_2 _= 2.460 (characteristic frequency of proto-oncogene proteins)

The designed peptides (18 amino acids sequence):

1) f_1 _= 0.0322, φ_1 _= -1.641, f2 = 0.0537, φ_2 _= -2.460

A: NLNEPAWQTRDDDDDRFM

2) f_1 _= 0.0322, φ_1 _= -1.641

B: LNEPHAYWQCRRDDDDDD

3) f_2 _= 0.0537, φ_2 _= -2.460

C: EILEPAWQRDDDDDRQWK

4) f_1 _= 0.0322, φ_1 _= 1.641, f_2 _= 0.0537, φ_2 _= 2.460

D: DRMWKPEILGPHKYYWWY

5) f_1 _= 0.0322, φ_1 _= 1.641

E: DDDRFMQWAAPPEILINV

6) f_2 _= 0.0537, φ_2 _= 2.460

F: CWAHEILEPAWQRDDDDD

It is known that proto-oncogene proteins are the products of proto-oncogenes. Generally, they do not have oncogenic or transforming properties but are involved in the normal regulation or differentiation of cell growth. However, proto-oncogenes can promote cancer development only if they acquire new properties as a result of mutations at which point they are known as oncogenes. Most common cancers involve modification of certain proto-oncogenes. Determination of two distinct characteristic frequencies, which correspond to two different functions, i.e. normal cell growth (proto-oncogenes) and uncontrolled cell transformation (oncogenes), reveal that the RRM approach can assist in distinguishing between the oncogene and proto-oncogene activity of oncogene proteins. Thus, these results can lead to the conclusion that the RRM approach is capable of identifying "cancerous" features (frequencies) within the protein primary structures of the studied proteins. The RRM presents a rational approach in generating phenotypic diversity in proteins that will complement the existing, more random mutagenic approaches currently used in directed evolution, or the more time consuming approach based on the prior knowledge of a 3-D structure. The possibility to computationally calculate the frequencies and phases with the following possibility to produce the desired biological mutations and alterations in proteins would benefit the development of new non-invasive treatments and technologies. In addition, the *de novo *peptides designed on the basis of the oncogenic (cancerous) and proto-oncogenic (normal) frequencies determined within the RRM might be used in the development of new testing and treatment procedures for cancer. It is particularly important to note that the RRM approach is capable of identifying the common (general) cancerous characteristic and thus it may lead to the development of a vaccine for the treatment of a wider range of cancers.

## Conclusion

We have shown previously using the RRM concepts that digital signal processing methods can be used to analyse linear sequences of amino acids to reveal the informational content of proteins and determine functionally significant amino acids of the analysed proteins. This study extends the utility of the RRM procedures to oncogene and proto-oncogene proteins. It has been also shown that the RRM is capable of identifying the difference between oncogenes and proto-oncogenes and thus, identifying general "cancerous" features within oncogene protein primary structures. If such feature can be identified then it would be possible to validate unknown or modified proteins as well as relevant DNA sequences for their possible cancerous activity. In addition, as shown here, it is possible to design peptides which would have only this "cancerous" characteristic. Such peptides are predicted to carry the common characteristic of all oncogenes and thus, they can be used for the development of a vaccine, which could be then preventive for most kind of cancers.

Hence, once again, it has been shown that the RRM provides a new strategy to characterise and interpret the informational content of proteins, particularly oncogene proteins relevant to cellular transformation. It also provides the guidelines for a wide variety of protein and peptide structural manipulations, e.g., protein engineering by recombinant techniques could be undertaken based on the RRM predictions for the redesign of specific protein or peptide variants with modified biological properties. This novel prediction scheme can be used to facilitate the structure-function studies of different proteins and thus, could result in significant cost saving and improvement in current biotechnology and quality of new biomaterials.
